# A Total-variation Constrained Permutation Model for Revealing Common Copy Number Patterns

**DOI:** 10.1038/s41598-017-09139-8

**Published:** 2017-08-29

**Authors:** Yue Zhang, Yiu-ming Cheung, Weifeng Su

**Affiliations:** 1 Electrical and Information College of Jinan University, Zhuhai, China; 2 Department of Computer Science, Hong Kong Baptist University, Hong Kong SAR, China; 3 HKBU Institute of Research and Continuing Education, Shenzhen, China; 4 BNU-HKBU United International College, Zhuhai, China

## Abstract

Variations in DNA copy number carry important information on genome evolution and regulation of DNA replication in cancer cells. The rapid development of single-cell sequencing technology enables exploration of gene-expression heterogeneity among single cells, providing important information on cell evolution. Evolutionary relationships in accumulated sequence data can be visualized by adjacent positioning of similar cells so that similar copy-number profiles are shown by block patterns. However, single-cell DNA sequencing data usually have low amount of starting genome, which requires an extra step of amplification to accumulate sufficient samples, introducing noise and making regular pattern-finding challenging. In this paper, we will propose to tackle this issue of recovering the hidden blocks within single-cell DNA-sequencing data through continuous sample permutations such that similar samples are positioned adjacently. The permutation is guided by the total variational norm of the recovered copy number profiles, and is continued until the total variational norm is minimized when similar samples are stacked together to reveal block patterns. An efficient numerical scheme for finding this permutation is designed, tailored from the alternating direction method of multipliers. Application of this method to both simulated and real data demonstrates its ability to recover the hidden structures of single-cell DNA sequences.

## Introduction

Profiling of genome-wide copy-number landscapes has been conducted by the application of next-generation sequencing technologies^1^. This strategy is popular for genotyping, and can include the comprehensive characterization of copy-number profiles by the generation of hundreds of millions of short reads in a single run^2^. Since next-generation sequencing uses bulk DNA from tissue samples, it provides an average signal from millions of cells, and is thus of limited utility for the characterization of tumor heterogeneity at the single-cell level.

Single-cell sequencing is a technique that has been developed to address key issues in cancer studies, including measurement of mutation rates, tracing of cell lineages, resolution of intra-tumor heterogeneity and elucidation of tumor evolution^3, 4^. Single-cell sequencing combines flow sorting of single cells, whole-genome amplification and next-generation sequencing to characterize the genome-wide copy number in single cells. Existing whole-genome amplification (WGA) techniques, such as degenerate-oligonucleotide-primed polymerase chain reaction^5^, multiple-displacement amplification^6^ and multiple-annealing looping-based amplification cycling^7^ inevitably introduce varying degrees of amplification bias when the whole genome of a single cell is amplified to microgram levels for next-generation sequencing^8, 9^. Besides, copy number profile detection requires only sparse sequence coverage^10^ and it makes a contribution to intrinsic noise of single-cell sequencing data. Technical noise that results from amplification bias is over-dispersed compared to Gaussian noises, and differs from the noise that occurs in bulk sequencing, which does not involve amplification.

There are four strategies that use next-generation-sequencing data to detect genome copy number, including read-depth, read-pair, split read and de novo assembly methods^11^. Read-depth-based methods are arguably most popular for detection of copy-number variation. The CNV detection method, circular binary segmentation (CBS)^12^, a statistical approach used in a single-cell sequencing protocol^13^, is a modification of binary segmentation to translate a noisy intensity read depth signal into regions of equal copy number. Copynumber^14^ combines least squares principles with a suitable penalization scheme for a given number of breakpoints and detects copy number profiles. Control-FREEC^15^ (control-free copy number and allelic content caller) uses least absolute shrinkage and selection operator (LASSO) regression to identify the breakpoint and detects copy number profiles. CNV-Seq^16^ (copy number variation using shotgun sequencing) uses a Gaussian distribution to model read depth signal. CNAseg^17^ (copy number abnormality segmentation) employs a hidden Markov model(HMM) and Pearson’s *χ* ^2^ statistic to segment read depth signal and get the copy number profile. Recent studies have shown that the intrinsic noise of read depth signal from single-cell sequencing data could be accurately characterized by negative binomial distribution^18^, NbCNV was then developed to reconstruct the copy number profile in single-cell DNA sequencing data^19^.

As single-cell-sequencing data accumulate, recent efforts have focused on simultaneously analyzing genome data from multiple samples to assemble and subgroup similar cells accurately and efficiently. This subgrouping will be helpful for the exploration of heterogeneity among single cells, providing important information in relation to cellular evolution. The simplest way to subgroup multiple samples into a block pattern is to sort their reads in ascending or descending order. However, the high levels of noise in single-cell-sequencing data negatively affect the reliability of sequence reads and lead to inaccurate patterns of variation. Matrix factorization is one of the most widely used techniques for finding a suitable representation of data in real applications by decomposing the expression measurements with linear basis matrix and its loading coefficients. Ideally, this representation reveals the latent structure in the data, and reduces the dimensionality of the data so that further computational methods can be applied^20^. A variety of methods have been developed to reveal hidden structures in observed data^21, 22^, such as social network clustering^23, 24^ and mining of protein or gene interactions^25, 26^. However, matrix factorization suffers from two drawbacks. Firstly, finding accurate linear basis and its spanning dimensions remains a challenging problem. Secondly, matrix factorization has a high computational cost, which limits its application to high-dimensional data analysis.

To directly recover block patterns within alignments of sequencing data from multiple single cells that might otherwise be obscured by noise, our aim is to develop an efficient method to re-alignment of the sequencing data. Principally, the proposed method involved continuous permutation of the data to achieve a pattern, in which similar samples are organized into clusters. This technique, named the total-variation constrained permutation (TCP) model, is based on the alternating direction method of multipliers. In TCP, the permutation is not stopped until the total variational norm of the recovered matrix is minimized, when similar samples are stacked together to reveal block structures.

The TCP method has two main advantages over previous methods. Firstly, the hidden structures are estimated directly, and little parameter pruning is needed. This high robustness facilitates its usage with various types of sequencing data with few manual interventions. Secondly, as the major operation involved is sample permutation, the TCP method, using the classic Kuhn-Munkres algorithm, is very efficient, greatly saving computational cost. This efficiency is particularly useful for the analysis of modern sequencing data, in which hundreds of thousands of signals need to be recovered.

## Results

Experiments were conducted to demonstrate the performance of the proposed TCP model. The test datasets included both synthetic and empirical single cell DNA sequencing data. The first experiment was designed to test the discriminatory power of the proposed method of TCP to group similar samples and form uniform patterns. Various levels of Gaussian noise were introduced artificially into the synthetic data to evaluate the robustness of the proposed TCP. In the second experiment, the TCP was tested on a single cell DNA sequencing data set of 100 breast-tumor cells to find their optimal organization, thus revealing block patterns.

### Experiments on Synthetic Data

#### Robustness and Accuracy Test over Noise Contaminations

**Figure 1:**
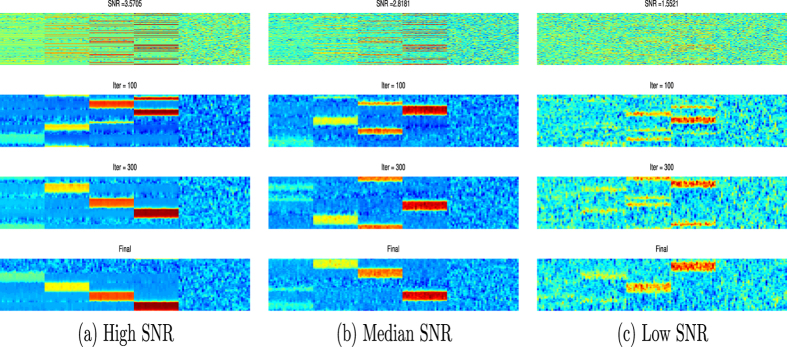
TCP analysis of a synthetic data set. A synthetic data set consisting of 100 samples is created, and Gaussian noise with zero mean value and (**a**) low, (**b**) intermediate, or (**c**) high variance is added to the data. The SNRs are (**a**) 3.57, (**b**) 2.82, and (**c**) 1.55. The raw observation is in the first row. The results of 100 and 300 iterations of TCP are shown in the second and third row, respectively, while the final result of TCP is shown in the last row.

A synthetic data set was created to test the permutation capability of the proposed TCP. The synthetic data consisted of 100 samples, drawn independently from four groups. The four groups were from different linearly expanded independent spaces and were created to be orthogonal. This configuration helped to create distinct block structure of the data. To test the robustness of the methods, Gaussian noise with zero mean value and three different variances was added to the data. The unmodified synthetic data are shown in Fig. 1(a), with an intermediate level of added noise in Fig. 1(b) and a high level of noise in Fig. 1(c). In each sub-figure of Fig. 1, each column (*x*-axis) is copy number reads along the genome, while each row (*y*-axis) is the sample. The synthetic data was drawn in heatmap and thus a larger value of copy number was hotter (red), while a smaller value of copy number was cooler (blue). The noise degradation was quantified by the signal-to-noise ratio (SNR). Figure 1 shows, from top to bottom, the results of TCP after 100 and 300 iterations, as well as the final result. When the noise level was low, TCP quickly organized the similar samples to group together, indicating the presence of four distinct sub-populations, highlighted in different colors. With an intermediate level of noise contamination, TCP still performed well, producing four distinct patterns. With a high level of noise contamination, TCP achieved some partial organization of the samples, but was not able to reproduce the four distinct patterns exactly. The high SNR patterns were accurately recovered, while the low SNR patterns were prone to be merged with the noises.

#### Recovery Uniform Pattern Test over Irrelevant Read Depth Signal

**Figure 2:**
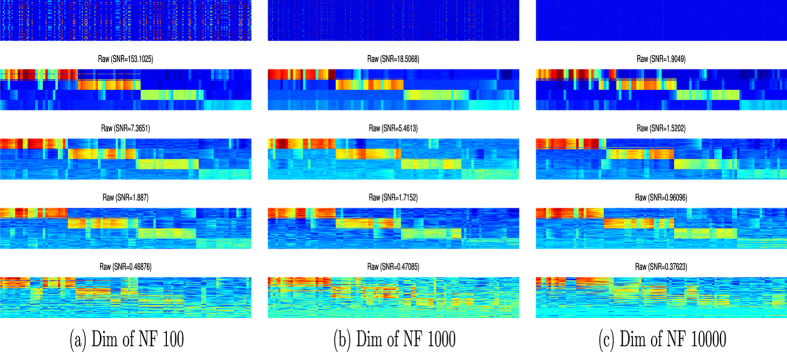
Recovery ability of TCP analysis. A synthetic data set consisting of 100 samples is created, and (**a**) 100, (**b**) 1,000, and (**c**) 10,000 artificial-read-depth signals are randomly inserted into the data. The raw data are shown in the top row. In each case, four levels of degraded noise are introduced as indicated by the SNRs, and the data are analyzed by TCP to recover uniform block patterns. In most cases, the four patterns are largely revealed by TCP in the presence of low or intermediate noise levels.

Real sequencing data usually contain information on millions of genes in each trial, thus non-zero copy numbers are very sparse if the normal copy number (two copies) is normalized to zero. To evaluate the potential of the proposed TCP in more general cases, artificial-read-depth signals were randomly inserted into each sample of the previously analyzed synthetic data. In this way, the length of the data was expanded to simulate real sequence data. The inserted signals were normalized to follow a normal distribution with a mean value of zero. The modified synthetic data therefore contain a large number of irrelevant, noisy features, completely burying the four distinct patterns. TCP analysis of the synthetic data set containing 100, 1,000 or 10,000 artificial-read-depth signals is shown in Fig. 2. The analysis was conducted with different degraded-noise levels (indicated by the SNR values). A feature-selection scheme was applied to remove irrelevant read depths signal before using TCP to group similar uniform pattern together. Feature selection used the Laplacian Score, which measures the importance of a read depth by its power of locality preserving and performs well in unsupervised data analysis^27^. This analytical result of a synthetic data set with addition of 100 noisy features is drawn by heatmap in Fig. 2(a). Similar to previous example, each column (*x*-axis) was the copy number reads while each row (*y*-axis) represent the profile of a sample. This addition had a minor influence on the performance of TCP compared with the noise level in the sample. As the noise level was increased, the four patterns became less discernable, as patterns with values below the noise level were not identified. Addition of 1,000 read depth signals (Fig. 2(b)) produced a similar result to addition of 100 read depth signals (Fig. 2(a)), and the performance of TCP was still acceptable even with the addition of read depth 10,000 signals (Fig. 2(c)). In each case, the four patterns were largely revealed by TCP in the presence of low or intermediate noise levels, but pattern discrimination was unsatisfactory with a high level of noise.

#### Comparison of TCP with Popular Methods

TCP was compared with three popular methods: Sparse non-negative matrix factorization (NMF), hierarchical clustering (HC) and Quick Sort. NMF is an unsupervised method for finding an approximate factorization of non-negative data. It decomposes a non-negative data matrix into two non-negative factors, one of which is considered to be an informative but concise representation of the original data, whereas the other is the corresponding loading factor^28^. The first factor can be thought of as the basis for a hidden linear space, whereas the second conveys important meta-expression information within that hidden space. NMF has been successfully applied to data clustering, classification and dimension reduction^29–32^. To cater to real applications, various constraints are imposed either on the feature or its loading matrix, or even on both. For example, Sparse NMF incorporates NMF with sparseness constraints on both the loading and feature matrices^20^. In hierarchical clustering, the similarity of sample was measured by Euclidean distance. The Quick Sort algorithm is a classic sorting method for organization of samples in ascending (or descending) order. The scheme uses the idea of “divide-and-conquer” to decompose large sample sets into multi-level subsets. Sorting is conducted on each subset and the results are combined to produce an ordered set. The quick sort algorithm was applied on the dataset to have the ordered pattern.

**Figure 3:**
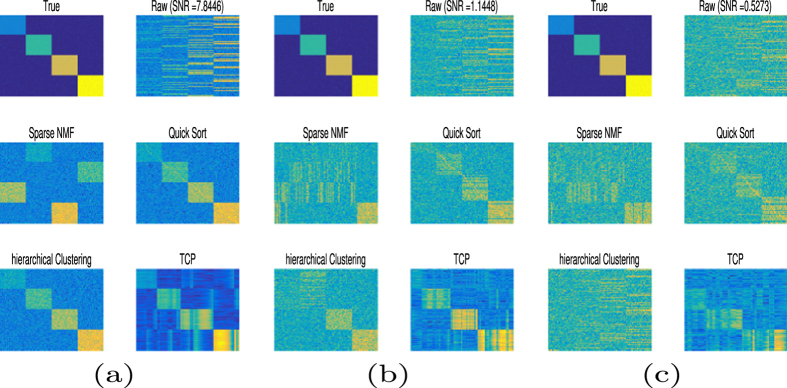
Performance comparison of TCP with the other methods. TCP is compared with the Sparse NMF, Quick Sort and hierarchical clustering methods on synthetic data under contamination with (**a**) low noise, (**b**) intermediate noise, and (**c**) high noise, as demonstrated by the SNRs of the raw data. TCP shows a high level of robustness to noise degradation, recovering the four distinct patterns in the true signal under all three noise levels.

To compare Sparse NMF, Quick Sort, HC and TCP, they were applied to the synthetic data set with contamination by Gaussian noise with zero mean value and three different variances (Fig. 3). For each noisy scenario, the figure shows the true data with similar samples positioned together and four distinct patterns. The the same data with added noise was consider as raw observation. It was analyzed by Sparse NMF, Quick Sort, hierarchical clustering and TCP to find hidden patterns. With a low level of noise contamination (Fig. 3(a)), all four methods accurately revealed the four distinct patterns. With an intermediate level of noise (Fig. 3(b)), Sparse NMF did not discriminate the four patterns, whereas the other three did. The Quick Sort did not clearly distinguish one block, as its signal level was similar to the noise level. The HC mistakenly mixed two low value blocks. The TCP method clearly revealed four patterns. Moreover, the recovered pattern had clear boundaries to its background. Such nice contrast was due to the enforced sparsity constraints within TCP model. When the noise level was high (Fig. 3(c)), TCP outperformed the other methods, and was the only method that produced identifiable patterns, demonstrating that TCP is highly robust to noise degradation.

### Application of TCP to Empirical Single-cell-sequencing Tumor Data

To further assess the performance of the proposed method on empirical applications, a single-cell-sequencing data set of 100 cells was downloaded from NCBI and tested^3^. The sequencing samples were from high-grade (grade III), triple-negative (*ER* ^−^, *PR* ^−^, *HER*2^−^) ductal carcinomas (T10). The sequencing workflow for these 100 single cells consists of flow sorting of single nuclei, whole-genome amplification, library construction and sequencing on an Illumina Genome Analyzer^13^. The 100 Illumina runs generated a total of 1.1 × 10^9^ reads, 5.8 × 10^10^ base calls (33.3 Gb downloads in sequence read archive (SRA) format) and were thus of low coverage. The data preparation pipeline consists of four steps, reads mapping, duplicates removing, read depth(RD) computing and data normalization. After downloading the sequencing file from NCBI SRA, bowtie2^33^ alignment tool was employed to map the millions of short reads to the hg19 human reference genome^34^ and Samtools was employed to remove potential PCR duplicates. RD signal was computed based on bins of variable sizes along the whole genome and normalized by locally-weighted polynomial regression and linear interpolation based on the GC content in each bin^13^. The data have been used to study the evolutionary dynamics and population structure of tumors in order to have a comprehensive view of the evolutionary process occurring in individual tumor cells^3^. The cells have been analyzed by fluorescence-activated cell sorting (FACS), and they consist of 47 diploids or pseudodiploids (2N), 24 hypodiploids (1.7N) and 29 aneuploids (3N or 3.3N). The diploid or pseudodiploid cells, on the whole, have a small number of copy-number variations, whereas the hypodiploids have narrow deletions, while the aneuploids have numerous copy-number duplications^3^.

**Figure 4:**
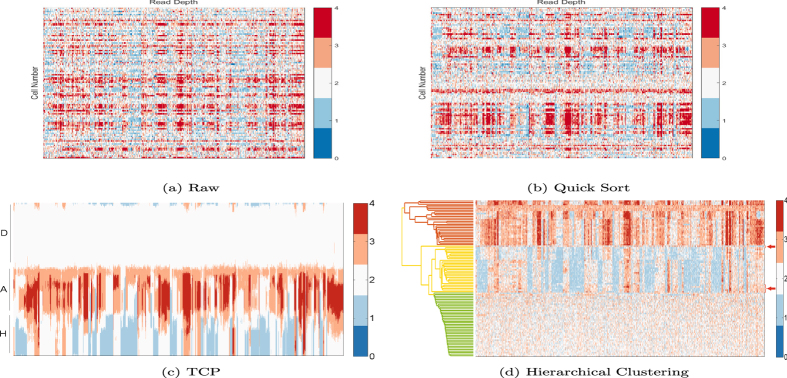
TCP analysis of empirical data. (**a**) The RD signals of 100 single-cell sequencing data; (**b**) result by quick sort scheme; (**c**) result after using the TCP to assemble similar samples at contingent positions; (**d**) result by hierarchical clustering. The data contains three subpopulations of (D) diploids or pseudodiploids; (A) aneuploids; and (H) hypodiploids. The result after TCP produces three blocks and is consistent with the previous characterization of the cells by FACS analysis^3^. From the top to the bottom, the ploidy of the clustered subpopulations in (**d**) is A, H, D, respectively. Five of the diploids or pseudodiploids are mis-classified to the hypodiploids by HC, as highlighted in arrow.

After the preprocessing steps, the normalized RD signals for the 100 cells were obtained. The TCP method was applied to the RD signals to reorganize their arrangement such that similar samples were in consecutive positions. For visual comparison, the correlative heat map of RD signals and the rearrangement after TCP are shown in Fig. 4. Each row represents the RD signals of a single cell. The columns represent contiguous segments of genome. Analysis of this data set by TCP reflected that the cells have three blocks as shown in (Fig. 4(c)) that were not apparent in the RD signals (Fig. 4(a)), or by simple sorting scheme (Fig. 4(b)). The three blocks corresponded to the “ploidy” groups identified by FACS analysis. The hypodiploid (1.7N) subgroup showed narrow deletions and the aneuploid (3N or 3.3N) subgroup showed rich duplications. The first five cells in the TCP output (represented by the top five rows in Fig. 4(c)) contained both deletions and duplications, but these variations could not be merged into the hypodiploid or aneuploid subgroups, which is consistent with the ploidy level (2N) of these five cells by FACS. For comparison, the result by hierarchical clustering is shown in Fig. 4(d). Hierarchical clustering divides the 100 single-cells into three subgroups. When inspecting the ploidy information in each cluster, it was found that hierarchical clustering misclassified five cells. The misclassified samples were highlighted in arrowed red slack parenthesis in Fig. 4(d).

## Discussion

With the accumulation of multiple single-cell-sequencing data, accurate and efficient subgrouping of similar cells remains a problem, because of the introduction of noise by the techniques required to accumulate enough start materials, and because of the huge genome lengths. Subgrouping will be helpful to explore gene-expression heterogeneity among single cells, providing important information on cellular evolution. We have now addressed this problem by developing a novel method for continually permutating of the data so that similar samples are positioned adjacently. The permutation is guided by the total variational norm of the recovered matrix. Since the major operation involved is sample permutation, the method, which uses the classical Kuhn-Munkres algorithm, is very efficient, greatly saving computational cost. Experiments on both simulated and real single-cell-sequencing data have demonstrated the superiority of the proposed method in recovering the hidden structures of samples, with few manual interventions.

## Methods

### Notation and Problem Statement

Throughout the paper, we let matrices be denoted by bold uppercase letters and vectors by bold lowercase letters. For a matrix ***X***, we use ***X***_*i*_ and ***X*** ^*j*^ to represent its *i*-th row and *j*-th column, respectively. For a matrix ***X*** ∈ *R* ^*m*×*n*^, its Frobenius norm is ${\Vert {\boldsymbol{X}}\Vert }_{F}=\sqrt{\sum _{i=1}^{m}\sum _{j=1}^{n}{x}_{ij}^{2}}$. The trace *Tr*(***X***) of a square matrix ***X*** is defined to be the sum of the elements on the main diagonal. The *l*_1_-norm for a vector ***x*** ∈ *R* ^*m*^ is defined by ${\Vert {\boldsymbol{x}}\Vert }_{1}={\sum }_{i=1}^{m}|{x}_{i}|$. A structural *l*_1_/*l*_*q*_-norm for matrix ***X*** is ${\Vert {\boldsymbol{X}}\Vert }_{{l}_{1},{l}_{q}}={\sum }_{j\mathrm{=1}}^{n}{\Vert {{\boldsymbol{X}}}^{j}\Vert }_{q}$. In particularly, the (*TV*, *l*_1_)-norm is the total variational norm with respect to each row of ***X***, i.e., ${\Vert {\boldsymbol{X}}\Vert }_{TV,{l}_{1}}={\sum }_{j}^{n}{\Vert {{\boldsymbol{X}}}^{j}\Vert }_{TV}$, where ${\Vert {{\boldsymbol{X}}}^{j}\Vert }_{TV}$ is defined as ${\Vert {{\boldsymbol{X}}}^{j}\Vert }_{TV}={\sum }_{i\mathrm{=2}}^{m}|{x}_{i,j}-{x}_{i-\mathrm{1,}j}|$.

Let ${\boldsymbol{B}}\in {{\mathbb{R}}}^{m\times n}$ represent a copy-number-variation profile dataset obtained from multiple samples, where *m* is the number of samples and *n* is the number of genes. Each entry (*i*, *j*) records the copy number at probe *j* of sample *i* and the value of (*i*, *j*) is denoted by *B*_*i*,*j*_. The *i*-th row ***B***_*i*_ corresponds to a copy-number profile obtained from sample *i*. We propose to use the following model to describe a given data set:

1 $${\boldsymbol{B}}={\boldsymbol{PX}}+\varepsilon $$

where ${\boldsymbol{X}}\in {{\mathbb{R}}}^{m\times n}$ denotes the permuted copy-number-variation signals by a permutation matrix ***P***, and *ε* is measurement noise, assuming a zero-mean normal distribution. We should note that although the technical noise resulting from amplification bias is over-dispersed than Gaussian noise does. For the simplify, we take the Gaussian noise for demonstration. A future study will be needed to assess the impact of non-Gaussian noise.

Our goal is to find an approximated matrix ***X***, in which similar copy-number signals are rearranged together. By the rearrangement, the estimated signal ***X*** could reveal the hidden-block characteristics accurately.

### Formulation

To make the decomposition in (1) feasible, we set up two assumptions:

**Assumption 1:** For each sample, the copy numbers are normalized around zero such that *B*(*i*, *j*) = 0 corresponds to the gain of two copy number at position *j* for the *i*-th sample. A copy number gain smaller than 2 (0 and 1) indicates deletion event, and copy number gain larger than 2 indicates duplication event. Thus the signal possesses sparsity.

**Assumption 2:** For a set of related samples, the copy number signals are likely to share similar patterns or linear correlation with each other.

The first assumption is generally valid in modern sequencing data because the variants, including single-nucleotide polymorphisms and copy-number variants, account for a small portion of the whole genome. The second assumption is the basic motivation for researchers to analyze multiple samples simultaneously.

Based on the above assumptions, we propose estimation of ***X*** by minimizing the following energy:

2 $$arg\,\mathop{\min }\limits_{{\boldsymbol{X}},{\boldsymbol{P}}}{\Vert {\boldsymbol{PX}}-{\boldsymbol{B}}\Vert }_{F}^{2}+\alpha {\Vert {\boldsymbol{X}}\Vert }_{TV,{l}_{1}}.$$

Here, we regularize ***X*** with a structural norm (*TV*, *l*_1_). The column-wise total variational norm aims to encourage the sample similarities, while the *l*_1_ norm aims to preserve the sparsity of copy number variants row-wise induced by the preprocessing step. Therefore, the regularization term satisfies the two pre-defined assumptions. *α* is a trade-off constant used to balance the data fidelity and structural similarity.

### Numerical Schemes

Minimization problems involving a total variational term are common in disparate areas, including signal processing and image recovery^35^. To obtain a sparse solution attributing to the total variational norm, a normal practice is to use the Lasso numerical method to solve the problem^36, 37^. However, a common drawback is the highly demanding computational cost resulting from the necessity to calculate a matrix inversion and the introduction of too many slack variables.

Recently, research results^35, 37^ have successfully demonstrated the efficiency of alternative direction of minimization (ADM) in solving image-restoration problems involving total variational norms. The numerical solution shows surprisingly fast speed and high accuracy. The image-restoration ADMs are variants of the classic Augmented Lagrangian method for optimization problems with separable structures and linear constraints, and they have been intensively studied in the optimization community. Inspired by this idea, we reformulated the problem in Eq. (2) into an optimization problem with favorably separable structures, thus enabling it to be efficiently solved using ADMs. The separable structure decomposition is particularly useful for sequencing-data analysis because of both its large dimensionality and the large amount of data involved. What is more, it dramatically reduces computational cost by overcoming the matrix-inversion burden required by Lasso and majority-minimization schemes^35, 37^.

Mathematically, let ***Y*** = ***DX*** with ***D*** being the first-order difference matrix, the minimization problem (2) could be equivalently rewritten as,

$$arg\,\mathop{min}\limits_{{\boldsymbol{X}},{\boldsymbol{P}}}{\Vert {\boldsymbol{PX}}-{\boldsymbol{B}}\Vert }_{F}^{2}+\alpha {\Vert {\boldsymbol{Y}}\Vert }_{{l}_{1},{l}_{1}}$$

subject to

$${\boldsymbol{Y}}={\boldsymbol{DX}}$$

The augmented Lagrangian of the minimization problem is given by:

3 $$ {\mathcal L} ({\boldsymbol{P}},{\boldsymbol{Y}},{\boldsymbol{X}})=\alpha {\Vert {\boldsymbol{Y}}\Vert }_{{l}_{1},{l}_{1}}+{\Vert {\boldsymbol{PX}}-{\boldsymbol{B}}\Vert }_{F}^{2}+\langle {\boldsymbol{\lambda }},{\boldsymbol{DX}}-{\boldsymbol{Y}}\rangle +\frac{1}{2}\gamma {\Vert {\boldsymbol{DX}}-{\boldsymbol{Y}}\Vert }_{F}^{2}$$

where the parameters *λ* and *γ* are Lagrangian multiples.

This new formulation enables it to be decoupled into two separate sub-problems with variables ***X*** and ***Y***, respectively. Therefore, it can be solved in an iterative manner:

**Step 1:** Find

4 $${{\boldsymbol{X}}}^{(k+\mathrm{1)}}=arg\,\mathop{min}\limits_{{\boldsymbol{X}}}\{{\Vert {\boldsymbol{PX}}-{\boldsymbol{B}}\Vert }_{F}^{2}+\langle \lambda ,{\boldsymbol{DX}}-{{\boldsymbol{Y}}}^{(k)}\rangle +\frac{1}{2}\gamma {\Vert {\boldsymbol{DX}}-{{\boldsymbol{Y}}}^{(k)}\Vert }_{F}^{2}\}.$$

It is equivalent to:

5 $$\begin{array}{rcl}{{\boldsymbol{X}}}^{(k+\mathrm{1)}} & = & {\rm{\arg }}\,\mathop{min}\limits_{{\boldsymbol{X}}}\{{\rm{Tr}}\{({\boldsymbol{X}}-{{\boldsymbol{P}}}^{T}{\boldsymbol{B}}{)}^{T}({\boldsymbol{X}}-{{\boldsymbol{P}}}^{T}{\boldsymbol{B}})\}+{{\boldsymbol{\lambda }}}^{T}{\rm{Tr}}\{{\boldsymbol{DX}}\}\\  &  & +\frac{\gamma }{2}{\rm{Tr}}\{({{\boldsymbol{X}}}^{T}{{\boldsymbol{D}}}^{T}{\boldsymbol{DX}}-2{{\boldsymbol{Y}}}^{{(k)}^{T}}{\boldsymbol{DX}})\}\}\\  & = & arg\,\mathop{min}\limits_{{\boldsymbol{X}}}\,{\rm{Tr}}\{{{\boldsymbol{X}}}^{T}{\boldsymbol{X}}-2{{\boldsymbol{B}}}^{T}{\boldsymbol{PX}}+{\lambda }^{T}{\boldsymbol{DX}}+\frac{\gamma }{2}{{\boldsymbol{X}}}^{T}{{\boldsymbol{D}}}^{T}{\boldsymbol{DX}}-\gamma {{\boldsymbol{Y}}}^{{(k)}^{T}}{\boldsymbol{DX}}\mathrm{\}}.\end{array}$$

It has an explicit solution:

$${\hat{{\boldsymbol{X}}}}^{{(k+\mathrm{1)}}^{\ast }}={\mathrm{(2}I+\gamma {{\boldsymbol{D}}}^{T}{\boldsymbol{D}})}^{-1}\mathrm{(2}{{\boldsymbol{P}}}^{T}{\boldsymbol{B}}-{{\boldsymbol{D}}}^{T}\lambda +\gamma {{\boldsymbol{D}}}^{T}{{\boldsymbol{Y}}}^{(k)}\mathrm{)}.$$

Matrix ***D*** is a circulant matrix and thus can be diagonalized by Fourier transform as ***D*** = ***F*** ^*T*^ ***KF***, where ***F*** is 2-D discrete Fourier transform, and ***K*** is a diagonal matrix containing the discrete Fourier transform coefficients of the difference operator ***D***. It follows that

6 $${\hat{{\boldsymbol{X}}}}^{{(k+\mathrm{1)}}^{\ast }}={{\boldsymbol{F}}}^{T}{\mathrm{(2}I+\gamma {{\boldsymbol{K}}}^{T}{\boldsymbol{K}})}^{-1}{\boldsymbol{F}}\mathrm{(2}{{\boldsymbol{P}}}^{T}{\boldsymbol{B}}-{{\boldsymbol{D}}}^{T}\lambda +\gamma {{\boldsymbol{D}}}^{T}{{\boldsymbol{Y}}}^{(k)})$$

**Step 2**: Find

$$\begin{array}{rcl}{{\boldsymbol{Y}}}^{(k+\mathrm{1)}} & = & {\rm{\arg }}\,\mathop{min}\limits_{{\boldsymbol{Y}}}\{\alpha {\Vert {\boldsymbol{Y}}\Vert }_{{l}_{1},{l}_{1}}+\langle {\boldsymbol{\lambda }},{\boldsymbol{D}}{{\boldsymbol{X}}}^{(k)}-{\boldsymbol{Y}}\rangle +\frac{1}{2}\gamma {\Vert {\boldsymbol{D}}{{\boldsymbol{X}}}^{(k)}-{\boldsymbol{Y}}\Vert }_{F}^{2}\}\\  & = & {\rm{\arg }}\,\mathop{min}\limits_{{\boldsymbol{Y}}}\{\alpha {\Vert {\boldsymbol{Y}}\Vert }_{{l}_{1},{l}_{1}}+\frac{\gamma }{2}{\Vert {\boldsymbol{Y}}-({\boldsymbol{D}}{{\boldsymbol{X}}}^{(k)}+\frac{{\boldsymbol{\lambda }}}{\gamma })\Vert }_{F}^{2}\}\end{array}$$

Its analytical solution could be estimated by point-wise soft thresholding:

7 $${{\boldsymbol{Y}}}^{(k+\mathrm{1)}}={S}_{\frac{\alpha }{\gamma }}({\boldsymbol{D}}{{\boldsymbol{X}}}^{(k)}+\frac{{\boldsymbol{\lambda }}}{\gamma })$$

The soft-thresholding operator *S*_*α*_(*x*) is defined by:

$${S}_{\alpha }(x)=\{\begin{array}{ll}x-\alpha , & {\rm{if}}\,x > \alpha \\ 0, & {\rm{if}}\,-\alpha \le x\le \alpha \\ x+\alpha , & {\rm{if}}\,x < -\alpha \end{array}$$

**Step 3:** Find

8 $${{\boldsymbol{P}}}^{(k+\mathrm{1)}}={\rm{\arg }}\,\mathop{min}\limits_{{\boldsymbol{P}}}{\Vert {\boldsymbol{P}}{{\boldsymbol{X}}}^{(k)}-{\boldsymbol{B}}\Vert }_{F}^{2}.$$

This is a so-called linear assignment problem. It is generally solvable by using the classical Kuhn-Munkres algorithm in *O*(*n* ^3^) time^38, 39^. Kuhn-Munkres provided a permutation matrix to capture the relative ordering between a set of raw observations and the re-organized version of that set.

The above three steps are updated iteratively until converge to achieve the final solution of ***X*** and the optimal permutation matrix ***P***.

The prominent characteristic of the aforementioned numerical solution is that its separable structure enables rapid identification of a solution. After simple algebraic operations, explicit analytical solutions for the two sub-problems, Eqs (4) and (7) are obtained. In both of the sub-problems, the solutions involve low-cost calculations, which can be solved efficiently.

### Complexity Analysis

The optimal solution is obtained by alternately updating the sequence of {***X*** ^(*k*)^, ***Y*** ^(*k*)^, ***P*** ^(*k*)^}, which are given in Eq. (6), (7) and Eq. (8), respectively. In Eq. (6), the inverse of diagonal matrix (2*I* + *γ* ***K*** ^*T*^ ***K***) costs *O*(*m*). The products of ***F***, ***F*** ^*T*^ with the inverse diagonal matrix need *O*(*mlogm*). Similarly, the product of (2***P*** ^*T*^ ***B*** − ***D*** ^*T*^ ***λ*** + *γ* ***D*** ^*T*^ ***Y*** ^(*k*)^) costs *O*(*mlogm*), too. Thus, the total computation cost in Eq. (6) is *O*(*mlogm*).

In Eq. (7), the soft thresholding operator ${S}_{\frac{\alpha }{\gamma }}({\boldsymbol{D}}{{\boldsymbol{X}}}^{(k)}+\frac{{\boldsymbol{\lambda }}}{\gamma })$ costs *O*(*mn*), where *n* is the sample size. In Eq. (8), estimation of the permutation matrix is achieved by the classical Kuhn-Munkres algorithm and it costs *O*(*n* ^3^). In total, the computational cost is *O*(*n* ^3^).

However, it should be emphasized that the computational cost of the proposed TCP is not high, because empirical sequence samples usually have small sample sizes. Besides, earlier techniques to find common patterns often suffered from the drawback of the “curse of dimension”. For example, the complexity of the popular technique of matrix factorization^21, 22^ is on the order of *O*(*n* × *k* × *m*), where *m* is the feature dimension and *k* is the dimension of the reduced space. This value is much larger than *O*(*n* ^3^) if $n\,\prec \,m$.
